# Radiation induced CNS toxicity – molecular and cellular mechanisms

**DOI:** 10.1054/bjoc.2001.2100

**Published:** 2001-09-01

**Authors:** C Belka, W Budach, R D Kortmann, M Bamberg

**Affiliations:** Department of Radiation Oncology, University of Tübingen, Hoppe-Seyler Str. 3,72076, Tübingen

**Keywords:** radiotherapy, CNS toxicity, molecular and cellular mechanisms

## Abstract

Radiotherapy of tumours proximal to normal CNS structures is limited by the sensitivity of the normal tissue. Prior to the development of prophylactic strategies or treatment protocols a detailed understanding of the mechanisms of radiation induced CNS toxicity is mandatory. Histological analysis of irradiated CNS specimens defines possible target structures prior to a delineation of cellular and molecular mechanisms. Several lesions can be distinguished: Demyelination, proliferative and degenerative glial reactions, endothelial cell loss and capillary occlusion. All changes are likely to result from complex alterations within several functional CNS compartments. Thus, a single mechanism responsible cannot be separated. At least four factors contribute to the development of CNS toxicity: (1) damage to vessel structures; (2) deletion of oligodendrocyte-2 astrocyte progenitors (O-2A) and mature oligodendrocytes; (3) deletion of neural stem cell populations in the hippocampus, cerebellum and cortex; (4) generalized alterations of cytokine expression. Several underlying cellular and molecular mechanisms involved in radiation induced CNS toxicity have been identified. The article reviews the currently available data on the cellular and molecular basis of radiation induced CNS side effects.   http://www.bjcancer.com © 2001 Cancer Research Campaign


*British Journal of Cancer* (2001) **85**(9), 1233–1239
© 2001 Cancer Research Campaign
doi: 10.1054/ bjoc.2001.2100, available online at http://www.idealibrary.com on IDEAL

http://www.bjcancer.com

Review

### Radiation induced CNS toxicity – molecular and cellular mechanisms

**C Belka, W Budach, RD Kortmann and M Bamberg**

Department of Radiation Oncology, University of Tübingen, Hoppe-Seyler Str. 3, 72076 Tübingen

**Summary** Radiotherapy of tumours proximal to normal CNS structures is limited by the sensitivity of the normal tissue. Prior to the development of prophylactic strategies or treatment protocols a detailed understanding of the mechanisms of radiation induced CNS toxicity is mandatory. Histological analysis of irradiated CNS specimens defines possible target structures prior to a delineation of cellular and molecular mechanisms. Several lesions can be distinguished: Demyelination, proliferative and degenerative glial reactions, endothelial cell loss and capillary occlusion. All changes are likely to result from complex alterations within several functional CNS compartments. Thus, a single mechanism responsible cannot be separated. At least four factors contribute to the development of CNS toxicity: (1) damage to vessel structures; (2) deletion of oligodendrocyte-2 astrocyte progenitors (O-2A) and mature oligodendrocytes; (3) deletion of neural stem cell populations in the hippocampus, cerebellum and cortex; (4) generalized alterations of cytokine expression. Several underlying cellular and molecular mechanisms involved in radiation induced CNS toxicity have been identified. The article reviews the currently available data on the cellular and molecular basis of radiation induced CNS side effects. © 2001 Cancer Research Campaign http://www.bjcancer.com

**Keywords**: radiotherapy; CNS toxicity; molecular and cellular mechanisms

Toxicity to CNS tissues is a constraint to radiotherapy in several locations. Complex therapy planning employing three-dimensional planning or intensity modulated radiotherapy may help to prevent damage to critical neural structures. Nevertheless, the effectiveness of radiotherapy is frequently limited by the tolerance of CNS structures.

Understanding the cellular and molecular mechanisms may help to develop strategies to either increase the radiation tolerance or to treat CNS alterations induced by irradiation.

Clinical responses of normal CNS tissues to whole brain irradiation of larger brain volumes may be classified as acute, subacute and late. Acute effects occur within 48 h or during the first week after single dose or fractionated irradiation respectively and are characterized by drowsiness, headache and emesis. Subacute effects occur after 6–10 weeks and are characterized by fatigability and somnolence and are mostly reversible. Findings resulting from late changes of the brain include a diffuse decline in cerebral function, with cognitive impairment being most prominent. In contrast to that, late radiation necrosis may be associated with focal neurological signs, seizures or symptoms of increased intracranial pressure and is more frequently seen after high dose irradiation of limited CNS volumes.

The precise delineation of pathomechanisms within the complex CNS is hampered by several problems:

1. Pathological data from humans are limited to cases with extensive clinical problems.
2. Data from animals cannot be correlated to clinical problems in humans.
3. In vitro systems only allow conclusions regarding a single cell system or at best two interacting cell systems.
4. The time course of late toxicity cannot be simulated using in vitro systems.
5. Feedback mechanisms may generate hen and egg problems.
6. The treated volume and the dose determine the pattern of late toxicity. Volume and dose effects cannot be simulated adequately in vitro.

The distinction of certain functional compartments in the brain helps to cope with several of the above mentioned problems. In this regard, three different target compartments within the CNS tissues may be separated:

1. Neurons
2. Glial cells
3. Vasculature.

The distinction is somewhat arbitrary since all compartments display strong interactions and other, alternative, compartments may also be discerned. However, since most data currently available deal with changes within one of these compartments the distinction allows a comprehensive review of the data.

The interaction between different compartments is underlined by pathological models distinguishing three different types of alterations within the spinal cord (Schultheiss et al, 1995): Type 1 lesions involve only white matter parenchyma. Type II lesions are predominantly vascular with all other changes judged to be secondary. Type III lesions display alterations to both, white matter and the vasculature.

A disadvantage of any attempt to explain CNS toxicity based on functional compartments is the fact that neither possible interactions nor the sequences become evident. Furthermore, some observations are not easily integrated in any compartment and can therefore be only described as isolated findings. Nevertheless, we

*Received 17 May 2001*
*Revised 30 June 2001*
*Accepted 24 July 2001*

*Correspondence to:* C Belka believe that bearing these shortcomings in mind, the use of compartment models helps to integrate the available results from molecular or cellular studies on radiation induced CNS toxicity.

### CELL BIOLOGY OF RADIATION INDUCED BRAIN TOXICITY

### Neuronal cells

Recent studies have shown that radiation can induce apoptosis in neurons in vivo in newborn animals (Gobbel et al, 1998). Since no such event was found in the CNS of adult rats (Li *et al*, 1996) a direct contribution to the radiation induced CNS toxicity is unlikely. Therefore, current models do not favour a role of direct damage to neuronal cells for the pathogenesis of radiation induced CNS toxicity.

### Glial cells and Demyelination

Oligodendrocyte brain stem cells are essential for the understanding of some models of radiation induced demyelination. The key cell for the generation of mature oligodendrocytes is the oligodendrocyte type 2 astrocyte cell (O-2A) (Raff et al, 1983). O-2A cells give rise to mature oligodendrocytes, required for the formation of myelin sheaths. O-2A cells also differentiate into type 2 astrocytes which are involved in the generation of the unique electrical properties at the ranvier nodes (Ffrench-Constant and Raff, 1986). The pattern of O-2A cell differentiation is regulated by a complex interplay of cytokines. A simplified model of O-2A differentiation is as follows (Figure 1): Embryonal O-2A cells which are transferred into serum free media are forced to differentiate prematurely into oligodendrocytes. However, if those cells are kept in PGDF medium or astrocyte conditioned medium they differentiate into oligodendrocytes following the same time schedule as they would do in the embryo (Noble et al, 1988; Raff et al, 1988). Adding serum and bone morphogenetic factor induces the differentiation into astrocytes (Mabie et al, 1997). The complementation of serum free media with PDGF and bFGF keeps the cell cycling in an undifferentiated progenitor stage. Most of the data on the differentiation pattern of O-2A cells are derived from embryonal O-2A cells. However, the adult brain also contains numerous O-2A cells which display only slight differences when compared to embryonal O-2A cells. Thus, the adult brain also contains an active glial stem cell compartment which might be a radiation target as well as an important source for CNS repair.

Only very limited data are available describing the behaviour of those cells during radiotherapy. Studies using rat optical nerves showed a decreased number of clonogenic O-2A cells after irradiation. In addition, the size of the colonies was found to be reduced, indicating a reduced clonogenic capacity of the surviving O-2A cells (van der Maazen et al, 1991a, 1991b).

Independently from the effects of radiation on progenitor cells, oligodendrocytes are deleted by radiation. In vitro studies showed that oligodendrocytes but not O-2A stem cells undergo apoptosis when being irradiated (Vrdoljak et al, 1992). A follow-up study showed that oligodendrocyte apoptosis also occurs in vivo after irradiation of rat spinal cord (Li et al, 1996). Furthermore, using p53 negative mice it was shown that radiation induced oligodendrocyte apoptosis in vivo is p53 dependent and executed by caspases (Chow et al, 2000).

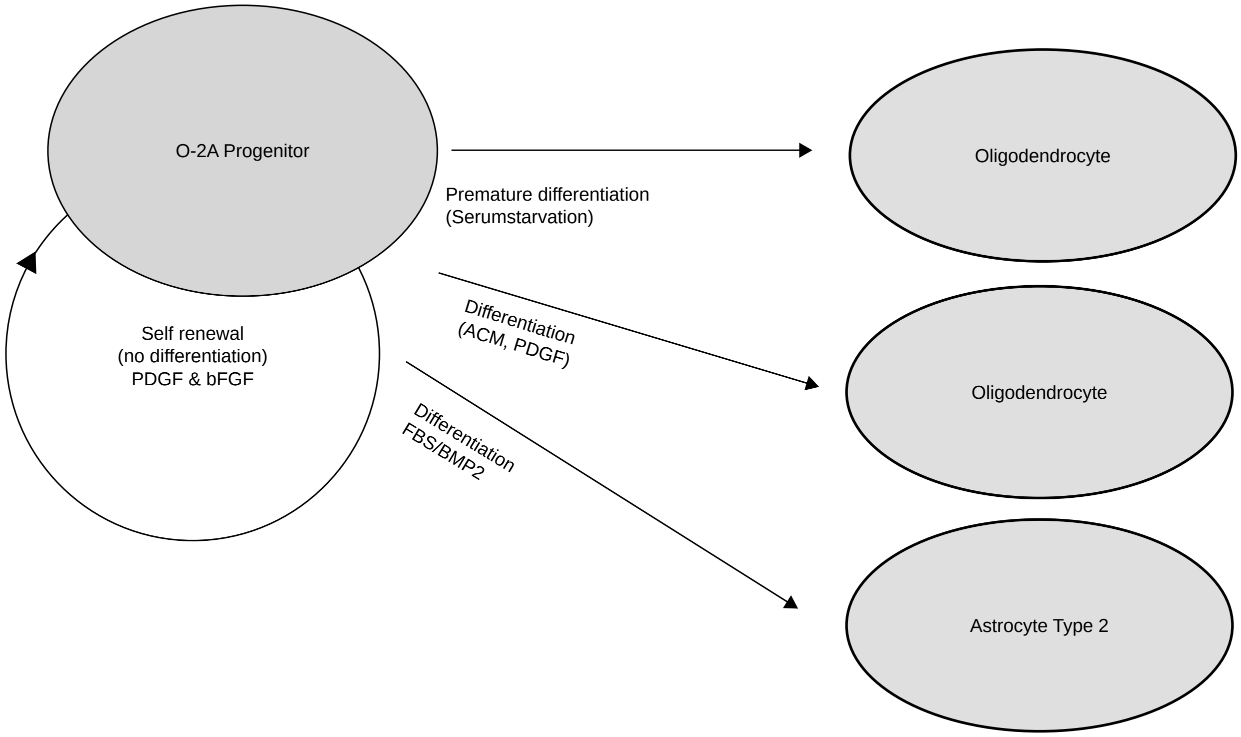

**Figure 1** Incubation of O-2A progenitor cells with platelet derived growth factor (PDGF) and basic fibroblast growth factor (bFGF) keeps the cells cycling in an undifferentiated stage. If O-2A cells are transferred into serum free medium they are forced to differentiate prematurely into oligodendrocytes. The addition of PDGF or astrocyte conditioned medium (ACM) induces differentiation into oligodendrocytes. In contrast, incubation of O-2A cells with fetal bovine serum (FBS) and bone morphogenetic factor (BMP) induces differentiation into type 2 astrocytes

 *© 2001 Cancer Research Campaign*

In addition to direct effects, radiation induced release of cytokines like TNF-α may exert additional toxicity to oligodendrocytes since it was shown that TNF-α induces cell death in those cells (Cammer, 2000).

Based on the data presented above, a speculative model of radiation induced demyelination could be as follows: Irradiation reduces the number of mature oligodendrocytes. Reductions in oligodendrocyte numbers trigger the recruitment of new oligodendrocytes from the O-2A compartment. In parallel, radiation directly effects the O-2A compartment in so far that both, the number of remaining O-2A cells and the capacity to form mature offspring is reduced. This speculative model explains two phases of the three phase time course of demyelination determined by the content of myelin basic protein (MBP) within the irradiated brain areas (Chiang et al, 1992). Guinea pig lumbar cords were irradiated and the MBP ratio inside to outside of the irradiated field was determined weekly. MBP was reduced at all time point after irradiation. However, a clear nadir occurred after 2–4 weeks, 7–8 weeks and around the onset of paralysis (11 weeks). These findings suggest that two reductions in myelin production are followed by an intermittent recovery and a final breakdown of myelin production. Assuming that myelin production reflects the numbers of oligodendrocytes this pattern suggests that two declines of myelin producing cells are counteracted by increased recruitment of new cells. However if the damage exceeds the stem cell reserve capacity toxicity results.

Nevertheless, several key issues remain unclear:

1. The contribution of the radiation mediated toxicity to O-2A cells and oligodendrocytes to the full picture of demyelination is unknown.
2. The contribution of secondary effects (i.e. altered cytokine expression or vascular damage) to demyelination is uncertain.
3. The relevance of demyelination for the clinical pictures of CNS toxicity is not defined.
4. The models derived form O-2A and oligodendrocyte responses in vitro are not fully compatible with the time course of demyelination in vivo.

### Toxicity to the vasculature

Alterations of the vasculature are of crucial importance for the development of CNS alterations in response to irradiation. Histological studies help to discern mechanisms of radiation induced vessel changes. Lifting of endothelia from the basement membrane, vacuolation of the cytoplasm and nuclear swelling are among the first effects seen and indicate that death of endothelial cells is an early event in small vessels which may be responsible for the initial oedema (Phillips, 1966; Zollinger, 1970). A specialized apoptosis mechanism with generation of intracellular ceramide via acidic sphingomyelinase may be crucial for endothelial cell apoptosis. Interestingly, the generation of ceramide can be abrogated by bFGF making bFGF a potential modulator of radiation induced vessel toxicity. (Pena et al, 2000). An alternative mechanism involves adhering, irradiated leukocytes which cause endothelial cell apoptosis through TNF (Eissner et al, 1995). Nevertheless it is not clear whether apoptosis or postmitotic death is the key mechanism for the denudation of vessels.

Early changes are followed by progressive loss of endothelia. Platelets adhere to exposed matrix leading to the formation of platelet clusters and thrombi (Verheij et al, 1994). This phase occurs within weeks and months and is characterized by vessels partially or fully occluded by thrombi. Subsequently, abnormal endothelial proliferation is observed. In parallel, thickening of the basement membranes and replacement of the lumen by collagen has been described (Adamson et al, 1970). However, the mechanisms regulating endothelial cell proliferation, basement membrane thickening and collagen production in response to ionizing radiation are not analysed in greater detail.

Possible cellular mechanisms involved in the pathogenesis of radiation induced vessel toxicity comprise the upregulation of diverse adhesion molecules. In this regard, the upregulation of E-selectin and ICAM-1 are of key importance (Quarmby et al, 1999). Both adhesion molecules were shown to be upregulated in response to radiation and mediate the adherence of leukocytes to vessel walls (Hallahan & Virudachalam, 1997; Quarmby et al, 1999). The importance of radiation induced ICAM-1 expression is underlined by the finding that inflammatory responses along vessels strictly require ICAM-1 (Hallahan and Virudachalam, 1997). Adhering leukocytes may increase the vessel toxicity via TNF-α induced apoptosis (Eissner et al, 1995). Thus in addition to direct vessel toxicity by irradiation, a complex of secondary effects may aggravate the initial vessel toxicity.

### Toxicity to defined structural compartments of the brain

A second, more anatomically defined stem cell compartment in the brain has been described as potential radiation target. The so-called subventricular zone (SVZ) remains mitotically active into adulthood. Studies have shown that cells from this area have self-renewal capacity, differentiate into neurons or glia, migrate over longer distances throughout the brain and may be essential for repair processes after brain toxicity (Doetsch et al, 1999). Irradiation with doses of 2 Gy induces apoptosis in the subependyma of young rats (Hopewell and Cavanagh, 1972) (Bellinzona et al, 1996) and in proliferating cells of the hippocampus (Peissner et al, 1999) leading to a prolonged impairment of the repopulative capacity (Tada et al, 1999).

Similar to the situation with endothelial toxicity and glial cell toxicity, the exact role of toxicity to the SVZ stem compartment to the full picture of radiation induced CNS toxicity is unclear.

### MOLECULAR MECHANISMS INVOLVED IN RADIATION INDUCED CNS TOXICITY

### Apoptosis

#### *General apoptosis signal transduction*

As stated above the induction of apoptosis seems to be relevant for some extent of the radiation induced CNS toxicity. Therefore, the basic mechanisms of apoptotic signal transduction induced by radiation or death ligands will be introduced. Up to now two major apoptosis mediating cascades have been analysed in greater detail (Fig. 2). The first pathway is activated by members of the death ligand family including TNF-α, CD95-L, and TRAIL. The second pathway mediates apoptosis induced by DNA toxicity. Although an overlapping set of molecules is employed, the general signal transduction principles are different (Belka et al, 2000).

After activation of a death receptor (i.e. TNFα-R1, CD95, TRAIL-R1, TRAIL-R2) the adapter FADD mediates activation of caspase-8 (Belka et al, 2000). In case of TNF-α an additional

*© 2001 Cancer Research Campaign* 

linker molecule denoted TRADD is required for the recruitment of FADD (Hsu et al, 1996). In turn, caspase-8 triggers activation of downstream caspases including caspase-3. In parallel, caspase-8 also activates the pro-apoptotic BID molecule. BID triggers the release of cytochrome c from the mitochondria thereby activating caspase-9 via APAF-1. Similarly to caspase-8, caspase-9 activates downstream caspases including caspase-3 (Li et al, 1997).

In contrast to receptor mediated apoptosis, radiation induced apoptosis primarily relies on mitochondrial damage followed by caspase activation (Belka et al, 2000). P53 mediated activation of BAX may serve as paradigm for the stress activated mitochondrial apoptosis pathway (Miyashita and Reed, 1995). BAX was shown to associate with the permeability transition pore (PT pore) complex and, upon binding to this complex, BAX can induce release of pro-apoptotic molecules including cytochrome c and apoptosis inducing factor (AIF) (Narita et al, 1998) (Susin et al, 1999). The release of cytochrome c triggers the activation of caspase-9 via APAF-1 and dATP as stated above.

Although it has not been shown for all components mentioned above, several authors provided evidence that the key concepts of apoptosis regulation are also relevant for the brain (Chow et al, 2000; Dowling et al, 1996).

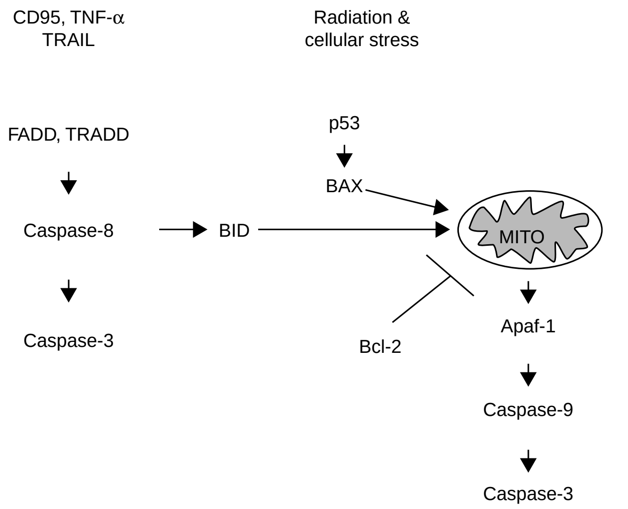

**Figure 2** Death receptor activation (CD95, TNF-α, TRAIL) stimulates downstream caspases via FADD and TRADD and the activator caspase-8. Via BID mitochondrial apoptosis may be activated. In contrast, DNA damage activates mitochondrial apoptosis pathways via p53 mediated BAX activation and release of cytochrome c. Cytoplasmic cytochrome c together with Apaf-1 and dATP activates caspase-9 and subsequently caspase-3. The anti-apoptotic protein Bcl-2 only interferes with the mitochondrial pathway

### ATM and apoptosis in the CNS

The product of the ATM gene (ATM) recently identified to be responsible for the ataxia telangiectasia (AT) phenotype is required for the regulation of apoptosis in some parts of the brain. ATM null mice display a strongly increased resistance towards radiation-induced apoptosis in certain anatomically defined regions of the brain (hippocampal denta gyrus, external granular layer of the cerebellum, retina). The lack of p53 conferred apoptosis resistance to the same regions of the brain indicating a mutual dependency of p53 and ATM for radiation induced apoptosis in the brain (Herzog et al, 1998). Another observation form the same group extended the knowledge regarding exact mechanisms of ATM regulated apoptosis in the brain showing an overlapping requirement for BAX and ATM in most regions of the brain. Furthermore the authors provided evidence that caspase-3 is the crucial executor caspase for radiation induced apoptosis in the brain (Chong et al, 2000).

### Stress induced gene expression

Parallel to the induction of apoptosis, the activation of gene expression seems to be of key importance for the development of CNS toxicity by ionizing radiation. As mentioned above, the increased expression of pro-inflammatory cytokines including TNF-α, INF-γ and adhesion molecules like ICAM participate in radiation induced brain toxicity. Thus, in order to understand the molecular mechanisms involved in radiation induced brain toxicity it is necessary to focus on signaling pathways involved in regulation of gene expression. Interestingly the regulation of TNF-α and ICAM-1 seems to involve overlapping pathways. For the purpose of comprehensibility the following paragraph will focus on pathways required for increased TNF-α gene expression.

Several studies provided evidence that the expression of TNF-α is directly activated by ionizing radiation (Hallahan et al, 1989). Unfortunately, in contrast to other stimuli only limited data are available describing pathways of TNF-α gene induction in response to radiation. Nevertheless, most of the data on TNF-α gene regulation revealed from other stimuli may also apply to radiation-induced gene regulation. Since the regulatory processes involved in TNF-α gene expression are highly complex, only key elements will be introduced.

Examination of the promoter region of the human TNF-α gene revealed both cell type-and stimulus-specific regulatory elements and potential binding sites for several transcription factors, including AP-1, NF-κB AP-2, NF-AT, SP-1, C/EBP, and cyclic adenosine monophosphate (cAMP) response elements (CRE). All of these transcription factors contribute to the complex regulation of the TNF-α gene. Of special importance regarding the radiation induced activation of the TNF-α gene as well as other genes are the transcription factors NF-κB, AP-1 and SP-1 which were shown to be inducible by ionizing radiation in general. A recent study also provided evidence that the induction of most of these factors also is detectable in irradiated brain tissue (Raju et al, 2000).

The transcription factor NF-κB, initially identified as regulator of the κB chain immunoglobulin gene, is crucial for the regulation of a multitude of human genes especially cytokine genes. In unstimulated cells, NF-κB proteins are sequestered in the cytoplasm by binding to inhibitor proteins, denoted 1-κBs. Binding of 1-κB to NF-κB masks the nuclear localization signal causing cytoplasmic retention. In response to stimulation, 1κB is phosphorylated and subsequently degraded. (Finco and Baldwin, 1995). A recently isolated kinase complex (IKK) which specifically phosphorylates 1κBs seems to be the key for activation of NF-κB. Activation of IKK is mediated by upstream kinases belonging to the stress activated kinase family for example MEKK1 (Lee et al, 1997) (Fig. 3).

The AP-1 transcription factor complex mainly consists of the c-jun and the c-fos protein and is activated by phosphorylation of c-jun through the stress activated protein kinase family. These kinases are part of a stress response network which is activated by most cellular stresses including ionizing radiation (Verheij et al, 1996). The paradigmatic stress cascade starts with the activation of the MEKK-1 kinase which phosphorylates the downstream kinase SEK-1 on serine residues. Activated SEK-1 induces the

 *© 2001 Cancer Research Campaign*

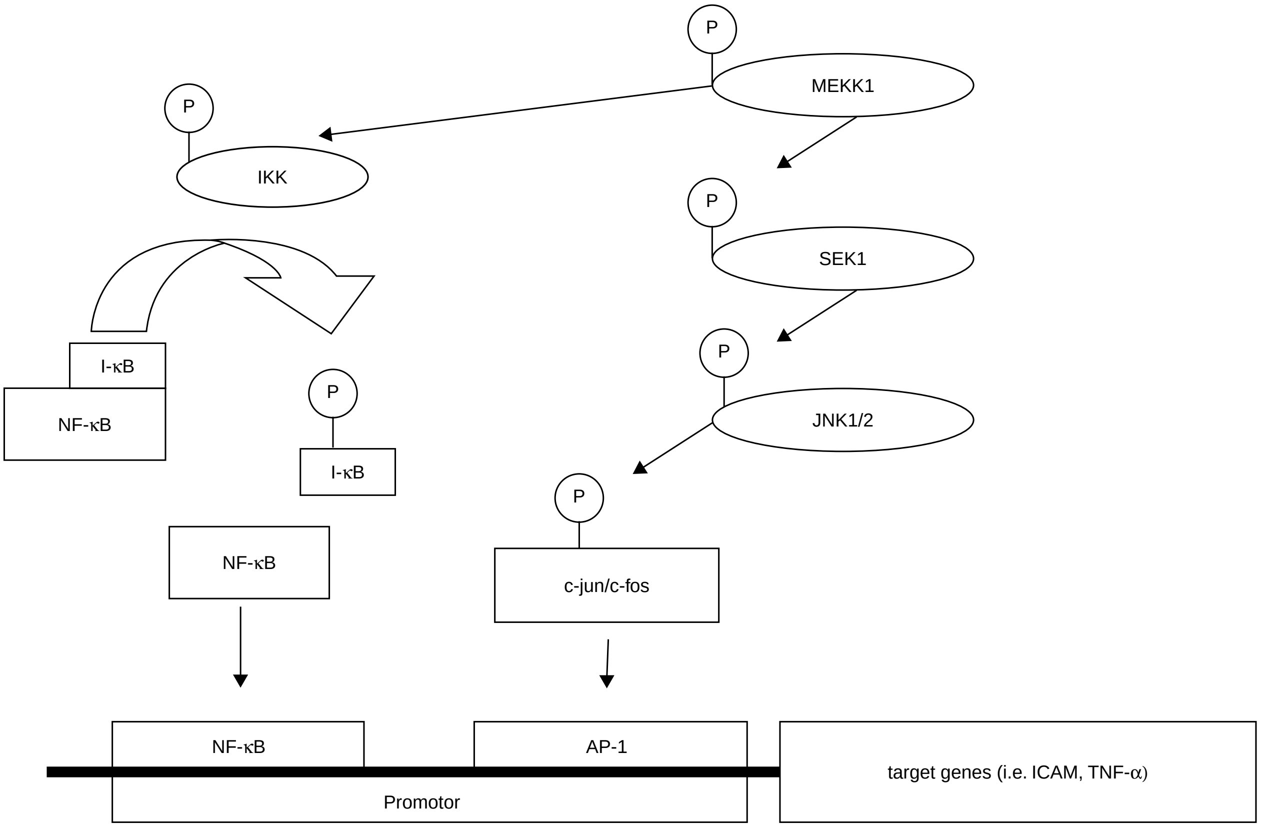

**Figure 3** Two basic mechanisms regulate gene expression in response to stress. Activation of NF-κB transcription factors is triggered by phosphorylation and degradation of I-κB. Degradation of 1-κB damasks the nuclear localization sequence of NF-κB allowing nuclear import. The phosphorylation of I-κB is mediated by a kinase complex (IKK) which is activated by kinases belonging to stress kinase pathway (MEKK-1). In parallel, stress activates the stress kinase pathway resulting in the activation of the transcription factor AP-1. Hallmark of this pathway is the signal transmission via subsequent phosphorylations. The most upstream kinase MEKK-1 activates SEK-1 which in turn phosphorylates and activates the jun-N terminal kinases (JNK1/2). These kinases phosphorylate the c-jun part of the AP-1 transcription factor and thereby strongly increase the transcriptional activity phosphorylation of JNK1/2 thereby triggering the activation of both kinases. Finally, JNK1/2 connect the kinase cascade to the activation of gene expression by phosphorylation of c-jun and ATF-2 (Sanchez et al, 1994) (Fig. 3). Despite the proven increase of SP-1 DNA binding activity in response to ionizing radiation, no data on the mechanisms of SP-1 regulation are available.

Only very few of the above mentioned regulatory processes have been analyzed in the CNS after irradiation. However the observation that key downstream elements of the stress response pathway, namely AP-1, NF-κB and SP-1 are activated by irradiation in vivo (Raju et al, 2000), suggests that the concept of the above mentioned stress response system also applies to the stress response in the CNS.

### PROPHYLAXIS AND TREATMENT – STRATEGIES AND OUTLOOK

A multitude of possible molecular targets to intervene with radiation induced CNS toxicity may be derived from the data presented (Fig. 4). However, only a few aspects have been experimentally approached yet. Thus it seems reasonable to believe that sooner or later radiation induced CNS toxicity may be ameliorated or become treatable.

Some of the most promising strategies are based on the use of stem cells. A basic study showed principally that retransplantation of purified O-2A cells into demyelinated lesions induced by ethidium bromide and radiation were remyelinated by O-2A cells (Groves et al, 1993). A recently identified source of therapeutic stem cells capable of myelinating lesions in the brain are totipotent embryonal stem cell which are differentiated into myelin-producing cells using PDGF, EGF and FGF2 (Brustle et al, 1999).

A different set-up using of cytokines was analysed by ljichi (ljichi et al, 1996). They tested in how far the transplantation of syngenic PDGF producing fibroblasts would increase the number of O-2A cells within the CNS and found that indeed the number of O-2A stem cells increased.

In addition to cell based strategies, several pharmacological approaches may prove effective for the prevention of radiation induced CNS side effects. Given the fact that stress induced gene expression is involved in the pathogenesis of CNS toxicity, any measures to block effector molecules (i.e. TNF-α/ICAM) or signaling molecules are suitable for the modulation of CNS toxicity. Several drugs blocking kinases (SB202190 for the p38 stress kinase), or transcription factors (MG-132 proteasome inhibitor for NFκB) are currently available. However, none of these strategies have been tested in animal models or patients.

Taken together, several molecular and cellular aspects of radiation induced CNS toxicity have been elucidated. Nevertheless, neither the precise role of individual mechanisms for the full picture of radiation induced CNS toxicity nor the best approach for treatment or prophylaxis have been determined yet.

© 2001 Cancer Research Campaign 

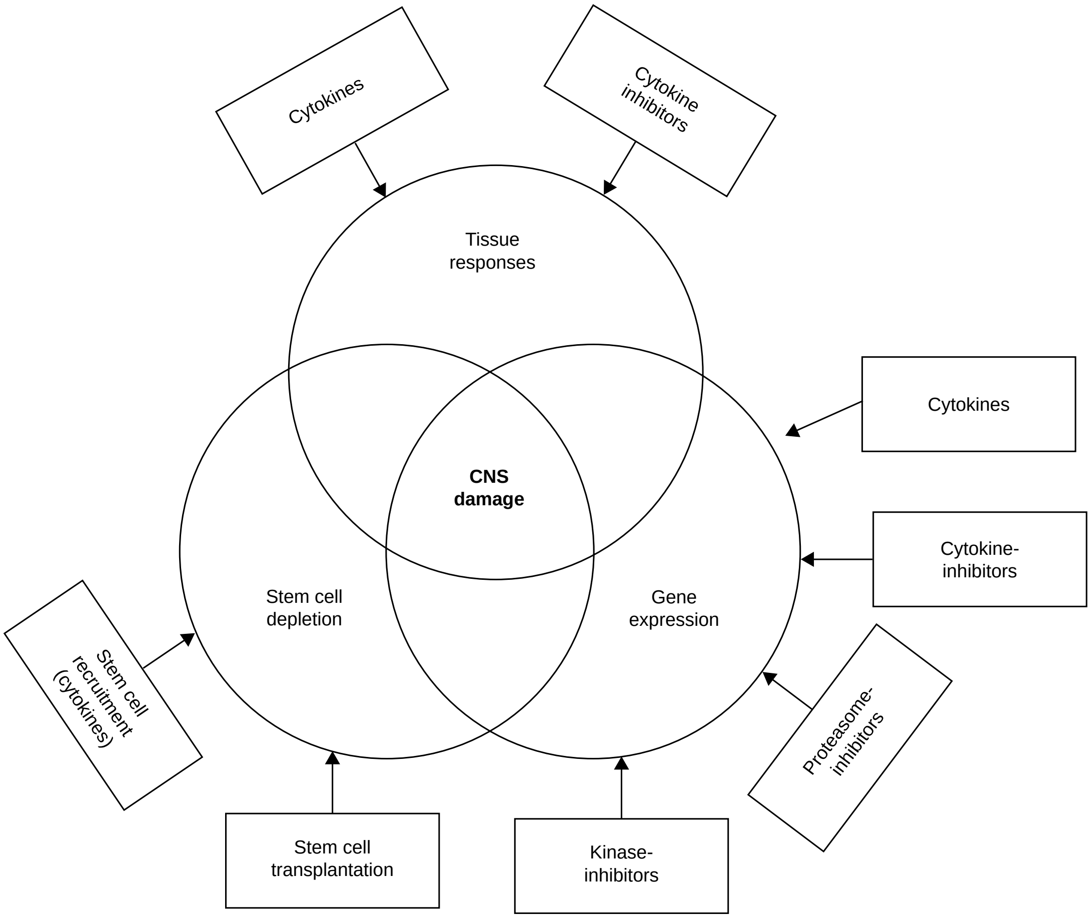

**Figure 4** From the data being available several targets for an intervention may be delineated. Gene expression which is involved in both, stem cell depletion and complex tissue responses may be influenced by kinase inhibitors, proteasome inhibitors, cytokines and cytokine inhibitors. Stem cell depletion may be corrected by direct application of purified stem cells or by cytokine mediated recruitment of stem cells. Complex tissue responses may be directly ameliorated by application of cytokines or cytokine inhibitors

### REFERENCES

Adamson IY, Bowden DH and Wyatt JP (1970) A pathway to pulmonary fibrosis: an ultrastructural study of mouse and rat following radiation to the whole body and hemithorax. *Am J Pathol* **58**: 481–498

Belka C, Rudner J, Wesselborg S, Stepczynska A, Marini P, Lepple-Wienhues A, Faltin H, Bamberg M, Budach W and Schulze-Osthoff K (2000) Differential role of caspase-8 and BID activation during radiation-and CD95-induced apoptosis. *Oncogene* **19**: 1181–1190

Bellinzona M, Gobbel GT, Shinohara C and Fike JR (1996) Apoptosis is induced in the subependyma of young adult rats by ionizing irradiation. *Neurosci Lett* **208**: 163–166

Brustle O, Jones KN, Learish RD, Karram K, Choudhary K, Wiestler OD, Duncan ID and McKay RD (1999) Embryonic stem cell-derived glial precursors: a source of myelinating transplants. *Science* **285**: 754–756

Cammer W (2000) Effects of TNFalpha on immature and mature oligodendrocytes and their progenitors in vitro. *Brain Res* **864**: 213–219

Chiang CS, Mason KA, Withers HR and McBride WH (1992) Alteration in myelin-associated proteins following spinal cord irradiation in guinea pigs. *Int J Radiat Oncol Biol Phys* **24**: 929–937

Chong MJ, Murray MR, Gosink EC, Russell HR, Srinivasan A, Kapsetaki M, Korsmeyer SJ and McKinnon PJ (2000) Atm and Bax cooperate in ionizing radiation-induced apoptosis in the central nervous system. *Proc Natl Acad Sci USA* **97**: 889–894

Chow BM, Li YQ and Wong CS (2000) Radiation-induced apoptosis in the adult central nervous system is p53-dependent. *Cell Death Differ* **7**: 712–720

Doetsch F, Garcia-Verdugo JM and Alvarez-Buylla A (1999) Regeneration of a germinal layer in the adult mammalian brain. *Proc Natl Acad Sci USA* **96**: 11619–11624

Dowling P, Shang G, Raval S, Menonna J, Cook S and Husar W (1996) Involvement of the CD95 (APO-1/Fas) receptor/ligand system in multiple sclerosis brain. *J Exp Med* **184**: 1513–1518

Eissner G, Kohlhuber F, Grell M, Ueffing M, Scheurich P, Hieke A, Multhoff G, Bornkamm GW and Holler E (1995) Critical involvement of transmembrane tumor necrosis factor-alpha in endothelial programmed cell death mediated by ionizing radiation and bacterial endotoxin. *Blood* **86**: 4184–4193

Ffrench-Constant C and Raff MC (1986) The oligodendrocyte-type-2 astrocyte cell lineage is specialized for myelination. *Nature* **323**: 335–338

Finco TS and Baldwin AS (1995) Mechanistic aspects of NF-kappa B regulation: the emerging role of phosphorylation and proteolysis. *Immunity* **3**: 263–272

Gobbel GT, Bellinzona M, Vogt AR, Gupta N, Fike JR and Chan PH (1998) Response of postmitotic neurons to X-irradiation: implications for the role of DNA damage in neuronal apoptosis. *J Neurosci* **18**: 147–155

Groves AK, Barnett SC, Franklin RJ, Crang AJ, Mayer M, Blakemore WF and Noble M (1993) Repair of demyelinated lesions by transplantation of purified O-2A progenitor cells. *Nature* **362**: 453–455

Hallahan DE, Spriggs DR, Beckett MA, Kufe DW and Weichselbaum RR (1989) Increased tumor necrosis factor alpha mRNA after cellular exposure to ionizing radiation. *Proc Natl Acad Sci USA* **86**: 10104–10107

Hallahan DE and Virudachalam S (1997) Intercellular adhesion molecule 1 knockout abrogates radiation induced pulmonary inflammation. *Proc Natl Acad Sci USA* **94**: 6432–6437

Herzog KH, Chong MJ, Kapsetaki M, Morgan JI and McKinnon PJ (1998) Requirement for Atm in ionizing radiation-induced cell death in the developing central nervous system. *Science* **280**: 1089–1091

Hopewell JW and Cavanagh JB (1972) Effects of X irradiation on the mitotic activity of the subependymal plate of rats. *Br J Radiol* **45**: 461–465

 *© 2001 Cancer Research Campaign*

Hsu H, Shu HB, Pan MG and Goeddel DV (1996) TRADD-TRAF2 and TRADD-FADD interactions define two distinct TNF receptor 1 signal transduction pathways. *Cell* **84**: 299–308

Ijichi A, Noel F, Sakuma S, Weil MM and Tofilon PJ (1996) Ex vivo gene delivery of platelet-derived growth factor increases O-2A progenitors in adult rat spinal cord. *Gene Ther* **3**: 389–395

Lee FS, Hagler J, Chen ZJ and Maniatis T (1997) Activation of the lkappaB alpha kinase complex by MEKK1, a kinase of the JNK pathway. *Cell* **88**: 213–222

Li YQ, Jay V and Wong CS (1996) Oligodendrocytes in the adult rat spinal cord undergo radiation-induced apoptosis. *Cancer Res* **56**: 5417–5422

Li P, Nijhawan D, Budihardjo I, Srinivasula SM, Ahmad M, Alnemri ES and Wang X (1997) Cytochrome c and dATP-dependent formation of Apaf-1/caspase-9 complex initiates an apoptotic protease cascade. *Cell* **91**: 479–489

Mabie PC, Mehler MF, Marmur R, Papavasiliou A, Song Q and Kessler JA (1997) Bone morphogenetic proteins induce astroglial differentiation of oligodendroglial-astroglial progenitor cells. *J Neurosci* **17**: 4112–4120

Miyashita T and Reed JC (1995) Tumor suppressor p53 is a direct transcriptional activator of the human bax gene. *Cell* **80**: 293–299

Narita M, Shimizu S, Ito T, Chittenden T, Lutz RJ, Matsuda H and Tsujimoto Y (1998) Bax interacts with the permeability transition pore to induce permeability transition and cytochrome c release in isolated mitochondria. *Proc Natl Acad Sci USA* **95**: 14681–14686

Noble M, Murray K, Stroobant P, Waterfield MD and Riddle P (1988) Platelet-derived growth factor promotes division and motility and inhibits premature differentiation of the oligodendrocyte/type-2 astrocyte progenitor cell. *Nature* **333**: 560–562

Peissner W, Kocher M, Treuer H and Gillardon F (1999) Ionizing radiation-induced apoptosis of proliferating stem cells in the dentate gyrus of the adult rat hippocampus. *Brain Res Mol Brain Res* **71**: 61–68

Pena LA, Fuks Z and Kolesnick RN (2000) Radiation-induced apoptosis of endothelial cells in the murine central nervous system: protection by fibroblast growth factor and sphingomyelinase deficiency. *Cancer Res* **60**: 321–327

Phillips TL (1966) An ultrastructural study of the development of radiation injury in the lung. *Radiology* **87**: 49–54

Quarmby S, Kumar P and Kumar S (1999) Radiation-induced normal tissue injury: role of adhesion molecules in leukocyte-endothelial cell interactions. *Int J Cancer* **82**: 385–395

Raff MC, Miller RH and Noble M (1983) A glial progenitor cell that develops in vitro into an astrocyte or an oligodendrocyte depending on culture medium. *Nature* **303**: 390–396

Raff MC, Lillien LE, Richardson WD, Burne JF and Noble MD (1988) Platelet-derived growth factor from astrocytes drives the clock that times oligodendrocyte development in culture. *Nature* **333**: 562–565

Raju U, Gumin GJ and Tofilon PJ (2000) Radiation-induced transcription factor activation in the rat cerebral cortex. *Int J Radiat Biol* **76**: 1045–1053

Sanchez I, Hughes RT, Mayer BJ, Yee K, Woodgett JR, Avruch J, Kyriakis JM and Zon LI (1994) Role of SAPK/ERK kinase-1 in the stress-activated pathway regulating transcription factor c-Jun. *Nature* **372**: 794–798

Schultheiss TE, Kun LE, Ang KK and Stephens LC (1995) Radiation response of the central nervous system. *Int J Radiat Oncol Biol Phys* **31**: 1093–1112

Susin SA, Lorenzo HK, Zamzami N, Marzo I, Snow BE, Brothers GM, Mangion J, Jacotot E, Costantini P, Loeffler M, Larochette N, Goodlett DR, Aebersold R, Siderovski DP, Penninger JM and Kroemer G (1999) Molecular characterization of mitochondrial apoptosis-inducing factor. *Nature* **397**: 441–446

Tada E, Yang C, Gobbel GT, Lamborn KR and Fike JR (1999) Long-term impairment of subependymal repopulation following damage by ionizing irradiation. *Exp Neurol* **160**: 66–77

van der Maazen RW, Kleiboer BJ, Verhagen I and van der Kogel AJ (1991a) Irradiation in vitro discriminates between different O-2A progenitor cell subpopulations in the perinatal central nervous system of rats. *Radiat Res* **128**: 64–72

van der Maazen RW, Verhagen I, Kleiboer BJ and van der Kogel AJ (1991b) Radiosensitivity of glial progenitor cells of the perinatal and adult rat optic nerve studied by an in vitro clonogenic assay. *Radiother Oncol* **20**: 258–264

Verheij M, Bose R, Lin XH, Yao B, Jarvis WD, Grant S, Birrer MJ, Szabo E, Zon LI, Kyriakis JM, Haimovitz-Friedman A, Fuks Z and Kolesnick RN (1996) Requirement for ceramide-initiated SAPK/JNK signalling in stress-induced apoptosis. *Nature* **380**: 75–79

Verheij M, Dewit LG, Boomgaard MN, Brinkman HJ and van Mourik JA (1994) Ionizing radiation enhances platelet adhesion to the extracellular matrix of human endothelial cells by an increase in the release of von Willebrand factor. *Radiat Res* **137**: 202–207

Vrdoljak E, Bill CA, Stephens LC, van der Kogel AJ, Ang KK and Tofilon PJ (1992) Radiation-induced apoptosis of oligodendrocytes in vitro. *Int J Radiat Biol* **62**: 475–480

Zollinger HU (1970) [Radiation vasculopathy]. *Pathol Eur* **5**: 145–163

© 2001 Cancer Research Campaign
